# Supplementary figures and images for: Transposition of HOPPLA in siRNA-deficient plants suggests a limited effect of the environment on retrotransposon mobility in Brachypodium distachyon
Source: PLoS Genet. 2024 Mar 12;20(3):e1011200. doi: 10.1371/journal.pgen.1011200 (PMC10959353; doi:10.1371/journal.pgen.1011200)

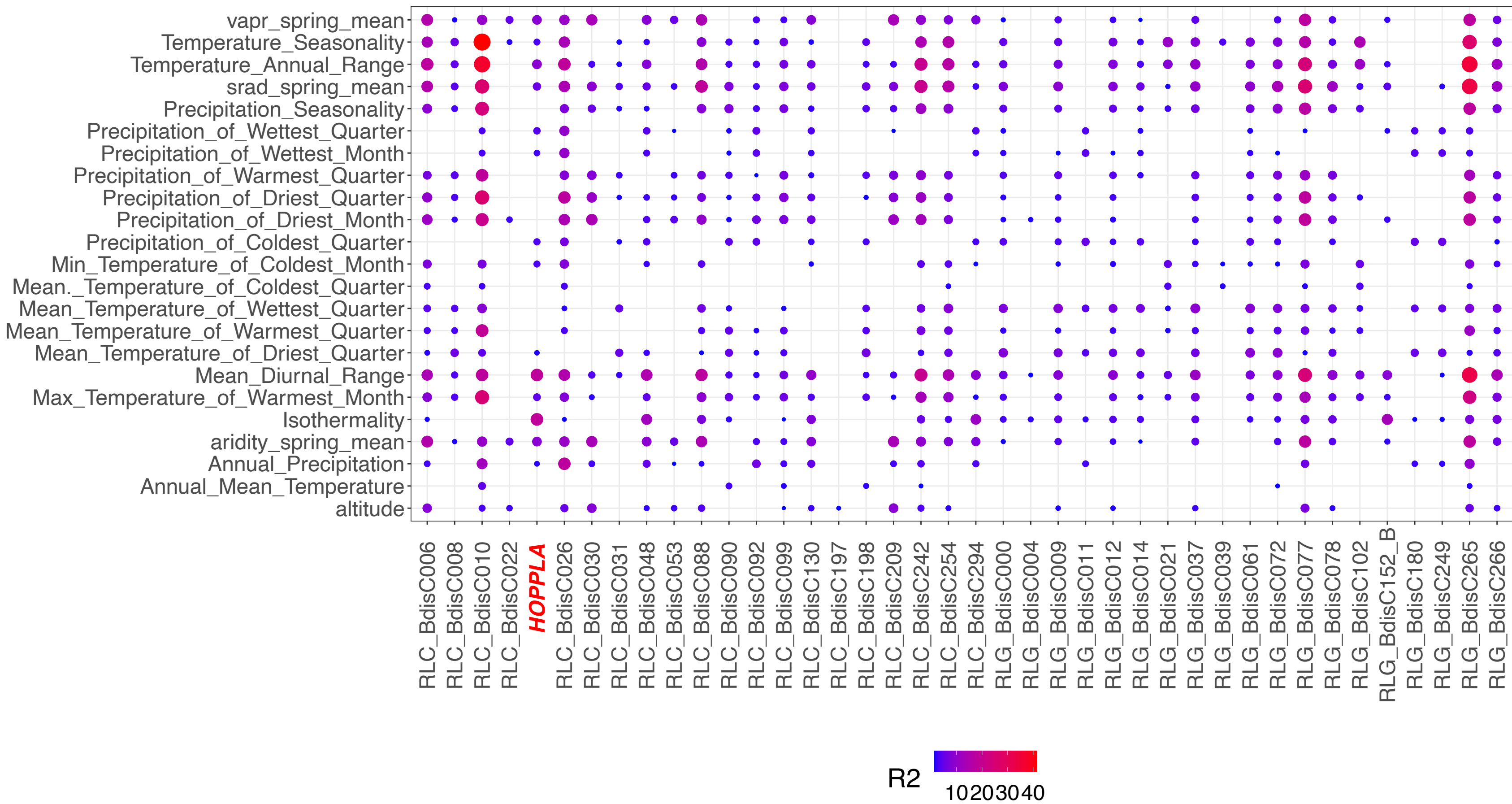

Supplement: S1 Fig — Colors and sizes of bubbles show the part of the variance (R2) explained by the bioclimatic variables in %. (PDF) [file pgen.1011200.s001.pdf]

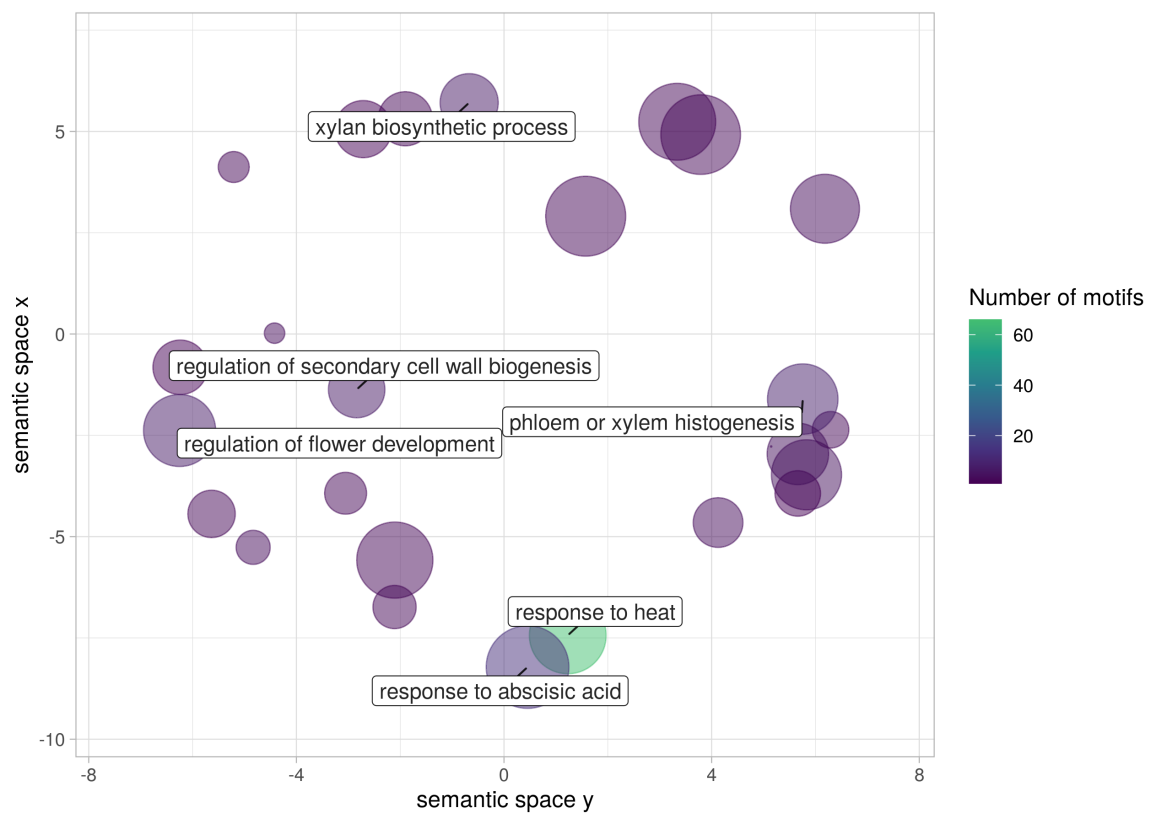

Supplement: S2 Fig — GO-enrichment analysis of transcription factors for which binding sites have been detected in AT1G11265, a member of the heat-responsive ONSEN (ATCOPIA78) LTR-RT family in A. thaliana. Colors indicate number of TF-binding sites found. GO terms that occur at least six times are highlighted in the plot. All GO-terms and their number of occurrences is listed in S1 Table. (PDF) [file pgen.1011200.s002.pdf]

**A**

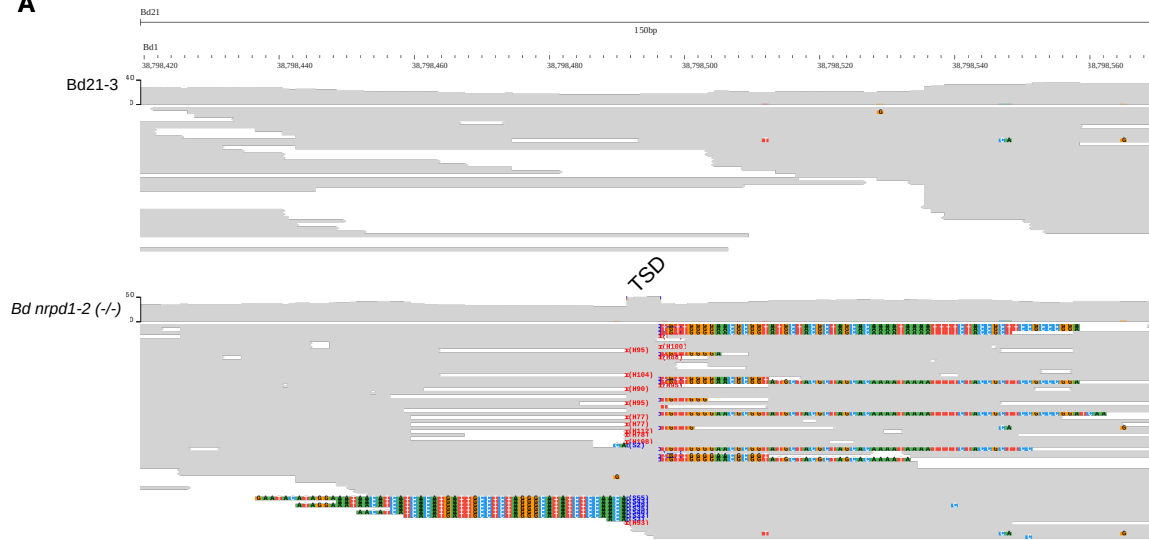

**B**

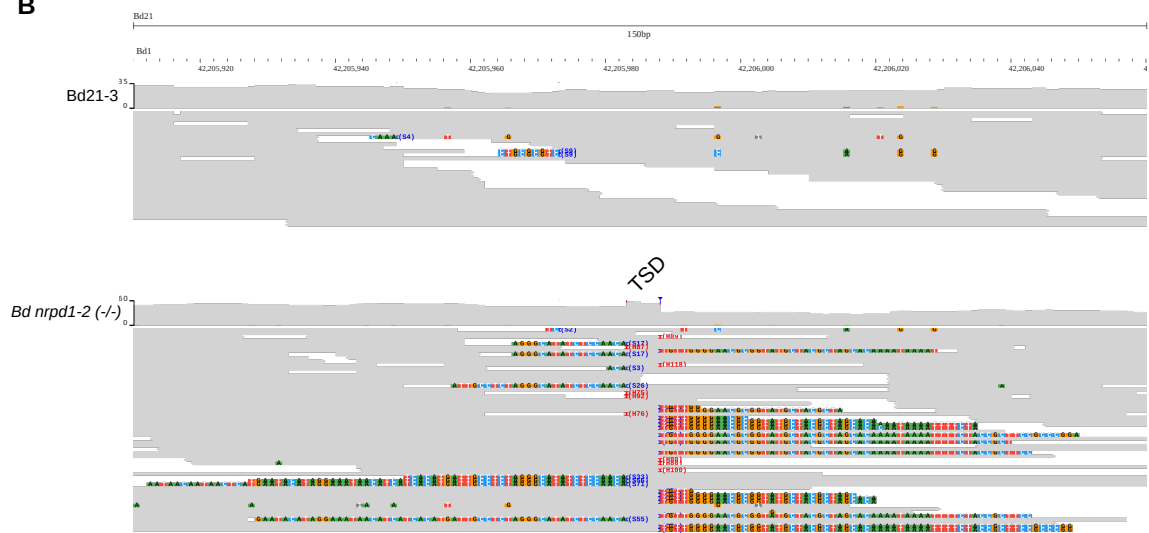

**C**

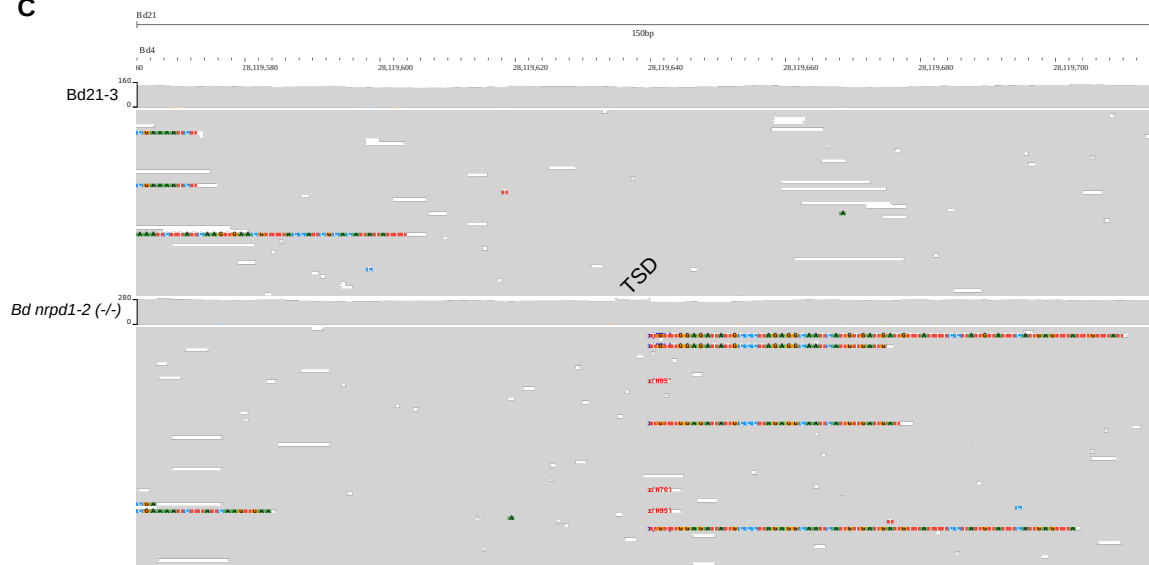

Supplement: S3 Fig — JBrowse screenshot of three insertion sites (A-C) in Bd nrpd1-2 (-/-) (bottom) compared to the Bd21-3 wt (top). The target side duplication (TSD) is annotated and soft clipped parts of reads are coloured. (PDF) [file pgen.1011200.s003.pdf]

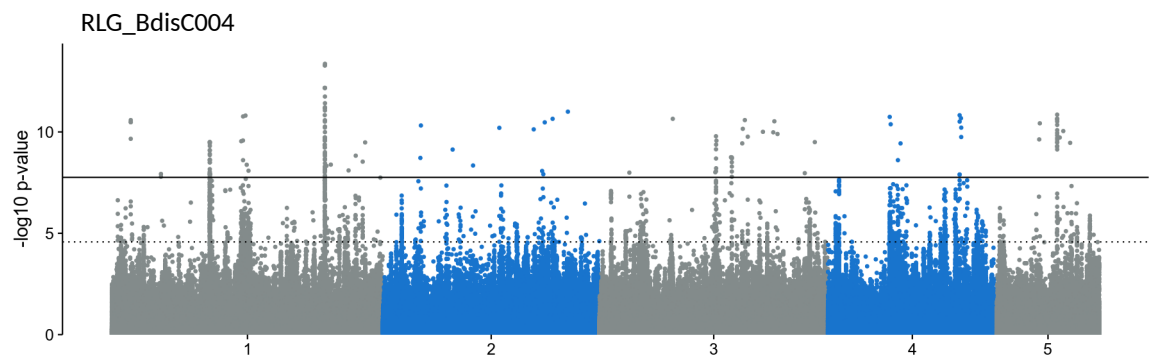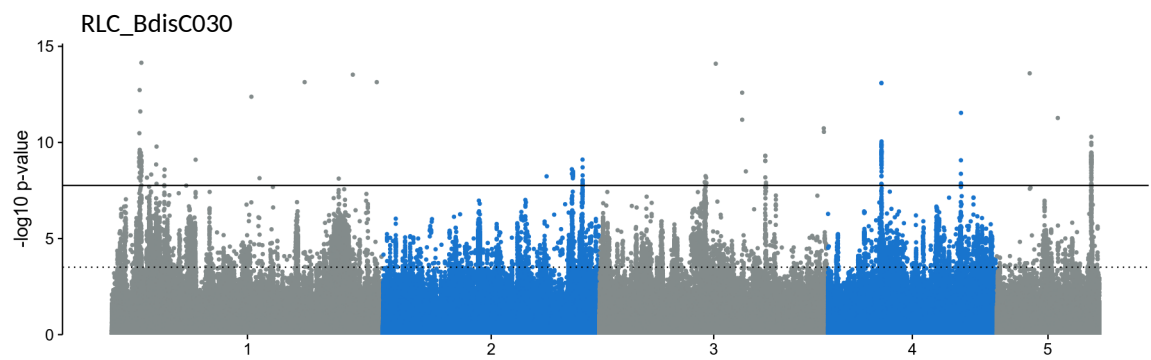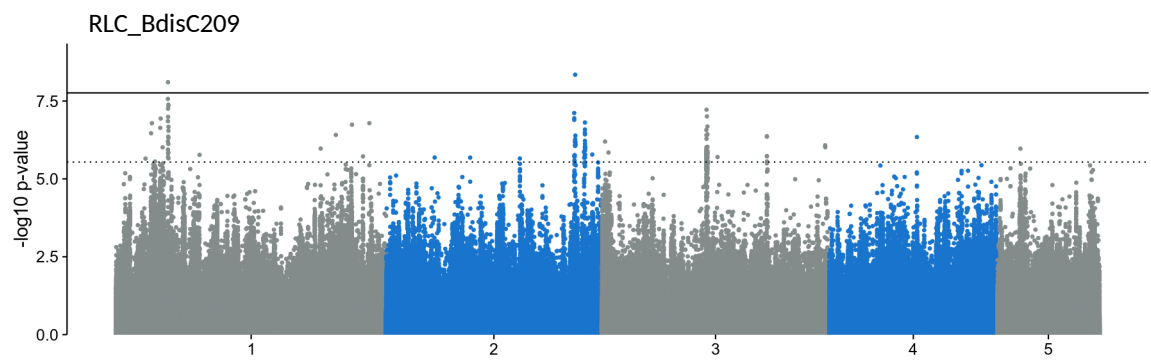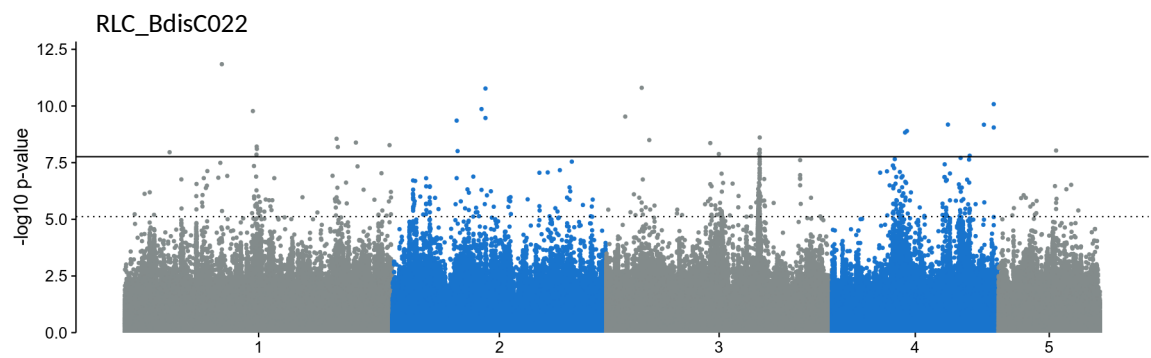

Supplement: S4 Fig — From top: RLG_BdisC004, RLC_BdisC030 RLC_BdisC209 and RLC_BdisC022. The two significance levels, false discovery rate < 0.05 (dashed line) and Bonferroni correction (solid line) are depicted. (PDF) [file pgen.1011200.s004.pdf]
